# Remote sensing monitoring and potential distribution analysis of *Spartina alterniflora* in coastal zone of Guangxi

**DOI:** 10.1002/ece3.11469

**Published:** 2024-05-31

**Authors:** Huanmei Yao, MeiJun Chen, Zengshiqi Huang, Yi Huang, Mengsi Wang, Yin Liu

**Affiliations:** ^1^ School of Resources, Environment and Materials Guangxi University Nanning China; ^2^ Key Laboratory of Environmental Protection (Guangxi University), Education Department of Guangxi Zhuang Autonomous Region Nanning China

**Keywords:** Landsat images, Maxent, phenology, potential distribution, random forest, *Spartina alterniflora*

## Abstract

In recent years, the continuous expansion of *Spartina alterniflora* (*S. alterniflora*) has caused serious damage to coastal wetland ecosystem. Mapping the coverage of *S. alterniflora* by remote sensing and analyzing its growth pattern pose great importance in controlling the expansion and maintaining the biodiversity of coastal wetlands in Guangxi. This study aimed to use harmonic regression to fit time series data of vegetation indices based on Landsat images, and the phenological features were extracted as the input of random forest model to distinguish *S. alterniflora* in coastal zone of Guangxi from 2009 to 2020. The influence of natural environmental factors on the distribution of *S. alterniflora* was evaluated by Maxent model, and the potential distribution was analyzed. The results showed that: (1) Based on the time series data of characteristic indices fitted by harmonic regression, the extraction of phenological features of *S. alterniflora* identification effect exhibited high accuracy (in the result of 2009, Overall Accuracy [OA] = 97.33%, Kappa = 0.95). (2) During 2009–2020, the *S. alterniflora* in coastal zone of Guangxi kept proliferating and expanding from east to west. The total area of *S. alterniflora* continued to increase while the growth rate showed a trend that increased first and then decreased. (3) The Maxent model shows good accuracy in simulating the habitat of *S. alterniflora*, with a potential distribution area of 14,303.39 hm^2^. The findings will be beneficial to the understanding of dynamic changes of *S. alterniflora* in coastal zone of Guangxi and provide a scientific reference for other coastal wetland studies on *S. alterniflora* expansion.

## Graphical abstract

In this paper, we employed harmonic regression analysis to fit time series data of vegetation indices based on Landsat 7/8 images and achieved the mapping of *Spartina alterniflora* in coastal zone of Guangxi from 2009 to 2020 by combining the extracted phenological features with random forest model. We used the Maxent model to evaluate the influence of natural environmental factors on the distribution of*S. alterniflora* and analyzed the potential distribution.

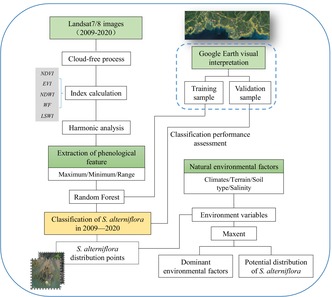

## 1 INTRODUCTION

**FIGURE 1:**
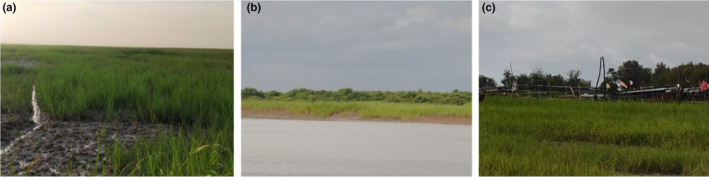
Field photographs of *Spartina alterniflora* (taken in the Dandou Sea).

*Spartina alterniflora* (Figure 1), a perennial herbaceous plant of the Poaceae family, was introduced to coastal regions of China in December 1979 with the aim of promoting sediment accumulation, mitigating wave erosion, safeguarding coastal beaches, and ameliorating soil properties (Tang & Zhang, 2003; Zhu & Qin, 2003). However, the rapid proliferation of *S. alterniflora* has resulted in accelerated sediment deposition, obstructing the flow of tidal channels and riverbeds. This not only disrupts wetland vegetation habitats but also poses a threat to the survival of intertidal organisms, thereby undermining the stability of wetland ecosystems (Chen et al., 2004; Wan et al., 2014). In recent years, *S. alterniflora*, listed as one of the invasive alien species in China, has been expanding in coastal areas of China, causing serious ecological problems (Jiang et al., 2022; Wang et al., 2006). The coastal zone of Guangxi is suffering from the expansion of *S. alterniflora* for its mild climatic conditions and wide tidal flat area, which provide a relatively suitable environment for the growth of *S. alterniflora* (Wu & Fang, 2005). In 1979, when first introduced to Dandou Sea, Hepu County, the coverage of *S. alterniflora* was only 0.67 hm^2^, but it reached an area of 304.21 hm^2^ in 2009 (Li et al., 2021). Since then, *S. alterniflora* has been widely distributed in Dandou Sea and gradually expanded to tidal flats in Tieshan Port and Yingpan Port. It has now proliferated in the tidal flats of the Nanliu River Estuary in Lianzhou Bay, showing a trend of continuous diffusion. The total area of *S. alterniflora* covered in the coastal zone of Guangxi reached 1159.21 hm^2^ by 2018 (Xu et al., 2021). The extensive growth of *S. alterniflora* along Guangxi's coastline encroaches upon valuable intertidal resources, squeezing indigenous wetland vegetation and disrupting salt marsh benthic communities. This poses threats to wetland ecosystem health and impacts coastal fisheries reliant on intertidal habitats (Deng et al., 2020). Effectively identifying *S. alterniflora*'s growth range, exploring its expansion patterns, and understanding its diffusion trends are crucial for restraining its spread, managing its growth areas, maintaining wetland ecosystem stability, and preserving coastal wetland biodiversity in Guangxi.

In recent years, some scholars have attempted to develop a spectral–phenological approach to identify *S. alterniflora*, achieving improved results (Tian et al., 2020). The accuracy of remote sensing recognition depends more on the features extracted. Ever since Li proposed the extraction of annual variation patterns of Normalized Difference Vegetation Index (NDVI) and Enhanced Vegetation Index (EVI) of *S. alterniflora* in coastal regions of Jiangsu based on the Moderate Resolution Imaging Spectroradiometer (MODIS) satellite images in 2006 (Li, 2006), numerous studies have been focusing on extracting phenological features. Previous research has demonstrated the applicability of time series phenological characteristics in identifying *S. alterniflora* (Sun et al., 2021; Wang, Wang, et al., 2021; Zhang et al., 2020), particularly in the senescence and green‐up stages, which better identify its coverage (Wang, Wang, et al., 2021). The previous studies proved that the phenological features extracted from time‐series data can accurately discriminate Spartina saltmarsh from other saltmarshes due to its significant seasonal variation in growth (Wu et al., 2020; Zhang et al., 2020). Landsat images, with their long temporal span and relatively high spatial resolution, can meet the accuracy requirements for long‐term observation of *S. alterniflora*. However, cloud disturbance and limitation in image quality result in the acquisition of few effective pixels for extracting phenological features from time series images, while the interference of pixel outliers significantly affects the data. To some extent, reconstructing time series data can remove noise and smooth curve data. Common methods are Savitzky–Golay (S–G) filter, Asymmetric Gaussian function fitting (AG) (Jönsson & Eklundh, 2002), Double Logistic (DL) (Beck et al., 2006), and Harmonic Analysis of Time Series (HANTS) (Gong et al., 2018). Harmonic regression, a data fitting method based on the discrete data Fourier transform (DFT) (Moody & Johnson, 2001), effectively analyzes periodically changing data. A study on fitting remote sensing image time series data using harmonic analysis has been conducted to calibrate NDVI data of different wetland vegetation by harmonic regression, demonstrating that it effectively reflects vegetation growth trends by eliminating high‐frequency noise while retaining low‐frequency data for fitting (Ashok et al., 2021; Lin & Mo, 2006) (Liang et al., 2011). Compared to other data fitting methods, harmonic regression exhibits higher fidelity and better smoothing effects.

The classification algorithms commonly used in remote sensing recognition include maximum likelihood method, support vector machine (SVM), decision tree classification, and Random Forest. Among these, Random Forest is widely utilized in wetland vegetation classification and integrates learning by combining multiple weak classifiers into strong classifiers. During the model construction process, input features and samples are randomly selected, leading to the advantages of random forest, such as good anti‐overfitting performance and strong robustness (Fu et al., 2017; Long et al., 2009; van Beijma et al., 2014; Wang et al., 2015). Eradicating *S. alterniflora* poses a challenge due to its rapid growth and high probability of recovery. Analyzing the spreading trend and potential distribution of *S. alterniflora* is of great significance for improving wetland ecosystem management and maintaining biodiversity in the coastal zone of Guangxi. The Maxent model (Phillips et al., 2006; Phillips & Dudik, 2008), one of the commonly used species distribution models, is based on the Maximum Entropy Principle and was proposed by the biologist Jaynes in 1957. The Maximum Entropy Principle implies that the subjective presumption of unknown conditions is excluded and all known conditions are used to limit the distribution probability of species, thus ensuring the greatest degree of stability of prediction analysis, achieving a stable model with the maximum entropy and the most uniform of probability distribution (Phillips et al., 2006; Wang et al., 2007). Studies have utilized the Maxent model to analyze potential species distribution, plan for species conservation, and analyze the correlation between species distribution and environmental variables. Some scholars have explored Maxent to explore the role of optimal bands in recognizing *S. alterniflora* and its crucial phenological stages (Liu et al., 2017). Others have compared the performance of different Species Distribution Models (SDMs), demonstrating that the Maxent Model's analysis effect is more precise and consistent (Kaky et al., 2020; Wang, Geng, et al., 2020), requiring fewer input samples and presenting better usability.

Since 2009, the growth range of *S. alterniflora* in the coastal zone of Guangxi has expanded from being predominantly distributed in Dandou Sea area to gradually encroaching on the tidal flats from Tieshan Port to Yingpan Port. It has now proliferated in the tidal flats at the mouth of the Nanliu River, showing an overall trend of continuous dispersion. In light of the considerations above, the coverage of *S. alterniflora* in coastal zone of Guangxi from 2009 to 2020 was identified in this study based on Landsat images. The specific objectives of this study are: (1) based on phenological features extracted from time series data fitted with harmonic regression, developing and evaluating a method capable of effectively classifying *S. alterniflora* in the coastal zone of Guangxi; (2) analyzing the spatial and temporal coverage of *S. alterniflora*, thus to better understand the expansion trend of the invasive species in the study area; and (3) using the Maxent model to analyze the potential distribution of *S. alterniflora*, providing scientific guidance for *S. alterniflora* management and monitoring.

## 2 MATERIALS AND METHODS

### 2.1 Study area

**FIGURE 2:**
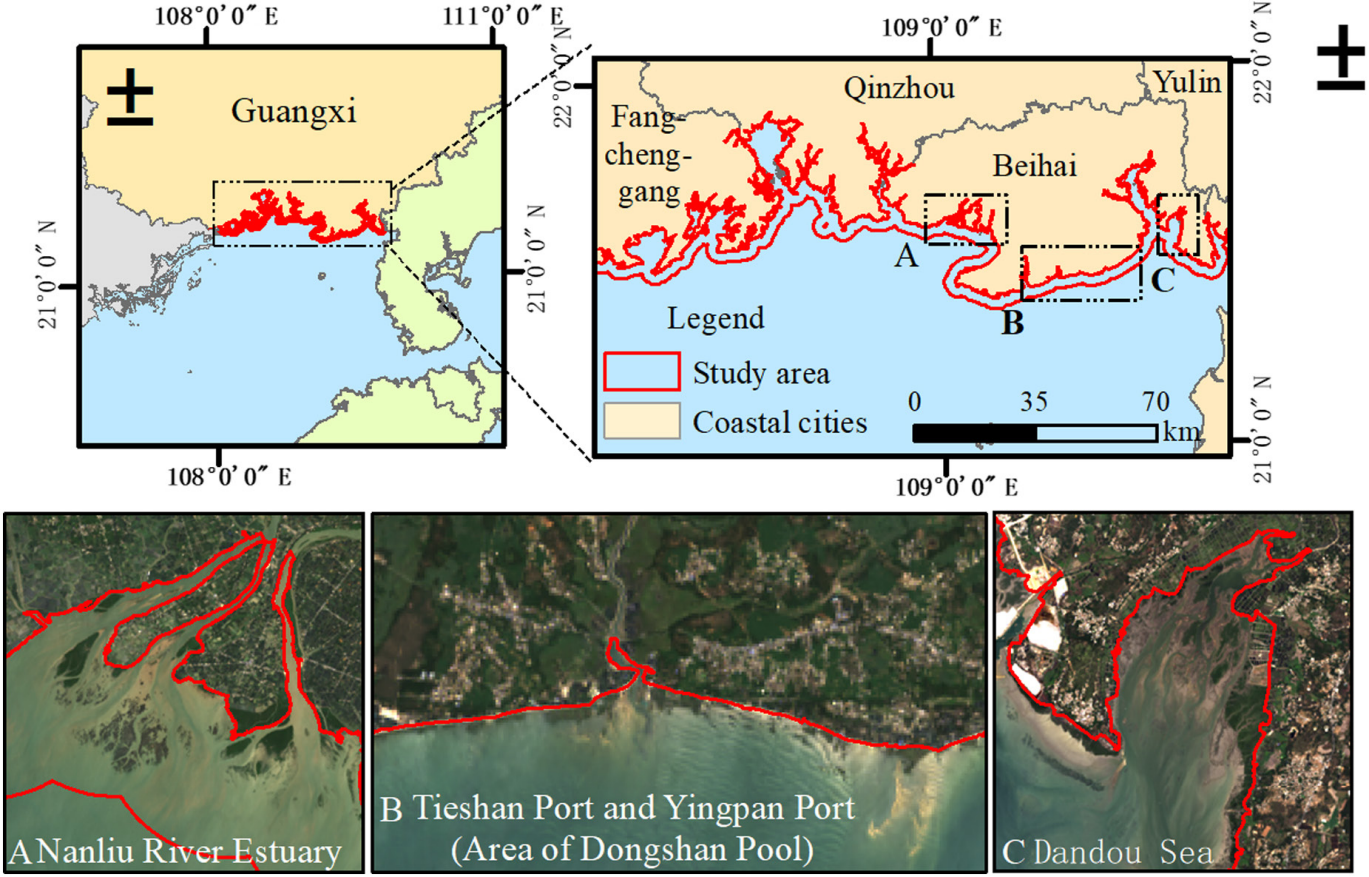
Study area.

The coastal zone of Guangxi is located along the mainland of Guangxi (107°57′ E ~ 109°48′ E, 21°00′ N ~ 22°15′ N), starting from the estuary of Beilun River at the junction of China and Vietnam in the west and ending at the estuary of Xiami River at the junction of Guangxi and Guangdong in the east. Being situated in the south of the Tropic of Cancer, the region has a subtropical marine monsoon climate characterized by the transition from subtropical to tropical. The mudflats in the coastal zone of Guangxi cover an area of 100,500 hm^2^, mostly distributed in Tieshan Port, Qinzhou Bay, and the estuary of Nanliu River (Long et al., 2009; Qi et al., 2018). Natural mangroves grow along the coast, with the largest coverage in China, mainly in Qinzhou Bay, the estuary of Nanliu River, Dandou Sea, etc. The expansion of *S. alterniflora* is crowding the living space of mangroves and other wetland vegetation to some extent. The study area is within 3 km of the coastline of Guangxi (Figure 2). *S. alterniflora* mainly grows in the estuary of Nanliu River, Tieshan Port, Yingpan Port, and Dandou Sea, with a small amount along Dafeng River. Therefore, it is urgent to assess the distribution of *S. alterniflora* to protect the biodiversity of native wetland vegetation.

### 2.2 Datasets and preprocessing

#### 2.2.1 Remote sensing data

The revisiting period of Landsat7/8 satellite is 16 days, and the spatial resolution is 30 m, which meets the accuracy requirement of *S. alterniflora* classification. Google Earth Engine (GEE) is a cloud platform for remote sensing data, where the acquisition and analysis of large‐scale geospatial data can be realized. The Landsat image data were obtained from the 2009–2020 Landsat 7/8 surface reflectance dataset (LANDSAT/LC08/C01 /T1_SR, LANDSAT/LE07/C01/T1_SR) archived in GEE (Masek et al., 2006; Vermote et al., 2016). Cloud‐free pixels were obtained by cloud mask processing according to the pixel quality assessment band (pixel_qa) in the dataset (Figure S1). All the Landsat image preprocessing tasks were carried out on the GEE platform.

The mangrove distribution data for 2020 derived from Landsat remote sensing images, which were distinguished by calculating the Mangrove Vegetation Index (MVI) (Baloloy et al., 2020) thresholds, and finally corrected by visual interpretation.

The spatial distribution data of tidal flats in 2020 are derived from the 10 m resolution China tidal flat spatial distribution dataset in the National Earth System Science Data Center (http://www.geodata.cn) (Jia et al., 2021).

#### 2.2.2 Training sample data

The training samples were obtained from Google Earth images from 2009 to 2020 with the resolution of 0.61–2.4 m.

Sample points of *S. alterniflora*, mangroves, tidal flats, rafts, and seawater were randomly selected in typical distribution regions within the study area as training samples. Training samples of different years were obtained from Google Earth images of corresponding years. To ensure a balanced and even distribution of training sample categories, the total number of training samples is not less than 550, with samples of each type not less than 100.

### 2.3 Methods

**FIGURE 3:**
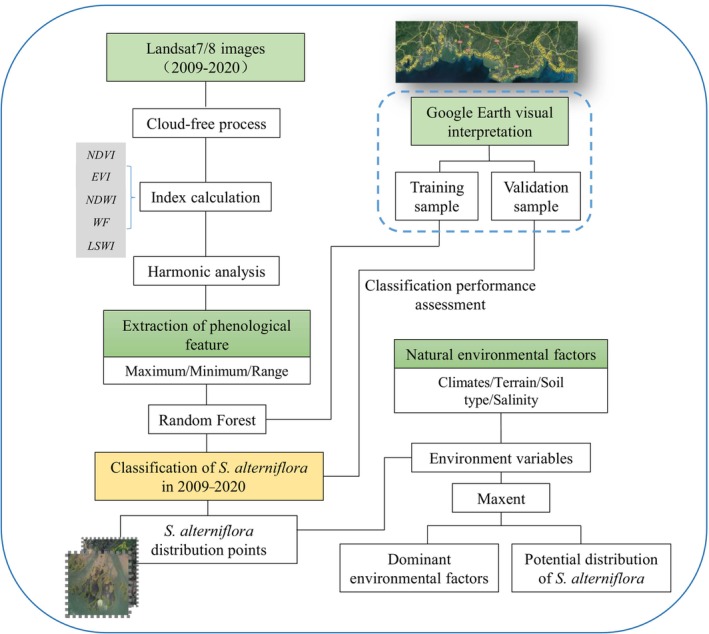
Workflow for mapping *Spartina alterniflora* in coastal zone of Guangxi.

In this study, the characteristic indices of each pixel in different years were calculated and fitted by harmonic regression. The extracted phenological features were used as the input of random forest and thus realized the classification of *S. alterniflora* in coastal zone of Guangxi. The potential distribution of *S. alterniflora* was analyzed by the Maxent model with the centroid of its identification results as the distribution point. The workflow for classification is shown in Figure 3.

#### 2.3.1 Extraction of phenological features

*Spartina alterniflora* is a salt marsh plant growing in the middle and upper part of the intertidal zone, with an obvious cycle change from growth to senescence, thus the extraction of phenological features can effectively distinguish *S. alterniflora* from another ground object. Considering the growth environment and species characteristics of *S. alterniflora*, Normalized Difference Vegetation Index (NDVI) (Tucker, 1979) and Enhanced Vegetation Index (EVI) (Huete et al., 2002) were selected as characteristic indices for vegetation classification, and Normalized Difference Water Index (NDWI) (Gao, 1996) was selected to distinguish water. Land Surface Water Index (LSWI) (Liu et al., 2020; Wang, Xiao, et al., 2020), which is more sensitive to vegetation moisture and soil moisture, was also selected as a characteristic index to distinguish wetland plants. Water Frequency (WF) is the ratio of the number of times that a pixel is recognized as a water body to the number of times that it is recognized as an effective pixel in a specific period. The selected characteristic indices are shown in Table 1.

**TABLE 1:** List of characteristic indices.

Characteristic indices	Formulas	Meaning	Features extracted
NDVI	$\frac{R_{\mathrm{NIR}}-R_{\mathrm{Red}}}{R_{\mathrm{NIR}}+R_{\mathrm{Red}}}$	Normalized Difference Vegetation Index	Maximum, minimum, and range
EVI	$\frac{2.5\times \left(R_{\mathrm{NIR}}-R_{\mathrm{Red}}\right)}{R_{\mathrm{NIR}}+6\times R_{\mathrm{Red}}-7.5\times R_{\text{Blue}}+1}$	Enhanced Vegetation Index	
LSWI	$\frac{R_{\mathrm{NIR}}-R_{\text{SWIR}1}}{R_{\mathrm{NIR}}+R_{\text{SWIR}1}}$	Land Surface Water Index	
NDWI	$\frac{R_{\text{Green}}-R_{\mathrm{NIR}}}{R_{\text{Green}}+R_{\mathrm{NIR}}}$	Normalized Difference Water Index	
WF	$\mathrm{WF}=\frac{N_{\text{water}}}{N_{\text{observation}}}$	Water Frequency	0 ~ 1

*Note*: *R*_red_, *R*_green_, *R*_blue_, *R*_NIR_, and *R*_SWIR_ are the reflectance of red, green, blue, near‐infrared, and short‐wave infrared bands, respectively, and *N*_water_ is the number of pixel identified as water (NDWI > 0 is determined as water); and *N*_observation_ is the number of available pixels in the same time period.

Due to the limitation of satellite revisiting period and image quality, there are fewer pixels available for *S. alterniflora* classification in the study area within a year. Due to the interference of solar altitude angle, observation angle, and atmospheric particulates in the process of image acquisition and processing, the time series data are prone to outliers, which can't show the original change of ground objects.

Harmonic represents time series data as a superposition of sinusoids in different frequencies, and a time component into frequency component (Lin & Mo, 2006). Building time series curve based on low‐frequency harmonic information, harmonic regression can eliminate the noise of high‐frequency harmonics to a certain extent, and the different expressed characteristic parameters at the same geographical location in different time periods can effectively reflect the actual change of ground cover (Liang et al., 2011). The formula is expressed as:

(1) $$y_{i}=a_{0}+\sum_{j=1}^{N–1}\left[a_{\mathrm{ij}}\cos\left(2\pi \omega \mathrm{jt}\right)+b_{\mathrm{ij}}\sin\left(2\pi \omega \mathrm{jt}\right)\right],i=1,2\ldots N$$

where *a*_0_ is the harmonic residual term; *a*_*ij*_ is the cosine coefficient; *b*_*ij*_ is the sine coefficient; *N* is the length of the time series; and *m* is the number of harmonics.

Based on the GEE platform, time series of NDVI, EVI, LSWI, and NDWI from 2009 to 2020 in the study area were constructed, respectively, by harmonic regression, and the maximum values, minimum values, and variation ranges of the indices were extracted as the input features of random forest model.

#### 2.3.2 Random forest model

Random forest is based on a CART (Classification and Regression Tree) that is a decision tree algorithm, whose core is to form a strong classifier from multiple weak classifiers.

The main process to construct a random forest model is as follows: (1) Based on the input features, different subsets of features are randomly selected as nodes to establish an independent decision tree. (2) Each decision tree is trained with training samples selected by bagging algorithm, and an independent set of classification rules are formed. (3) Independent decision results for validation samples are formed in different decision trees, and the final classification results are determined by voting. Due to the randomness in the selection process of samples and input features, the algorithm is highly resistant to overfitting and has stable performance (Breiman, 2001; Breiman et al., 2003).

#### 2.3.3 Accuracy assessment of the *S. alterniflora* maps

Based on the principle of stratified random sampling, validation samples were extracted from the classification results of *S. alterniflora* and non‐*S. alterniflora* by the “Create Accuracy Assessment Points” tool in ArcGIS 10.5. As the area of non‐*S. alterniflora* was much larger than that of *S. alterniflora* in the study area, with 150 points of *S. alterniflora* and 300 points of non‐*S. alterniflora* were extracted as validation samples. The vector files were converted into KML (Keyhole Markup Language) files and imported into Google Earth for visual interpretation. The Overall Accuracy (OA), Producer's Accuracy (PA), User's Accuracy (UA), and Kappa coefficients, calculated by the “Create Confusion Matrix” tool in ArcGIS 10.5, were used to assess the accuracy of the *S. alterniflora* classification results.

#### 2.3.4 Construction and execution of the Maxent model

Maxent niche model, which is based on the principle of maximum entropy and implemented by Java language, has been proven to be one of the most accurate SDMs of the maximum entropy theory available on the website of the American Museum of Natural History (https://biodiversityinformatics.amnh.org/open_source/maxent/).

In this study, the centroid of the *S. alterniflora* patches was extracted as the distribution points. The centroid refers to the center of gravity of the community calculated by the patch area weighting. The variation of *S. alterniflora*'s centroid can reflect its expansion direction and spatial changes over different years, providing insights for analyzing the expansion trend of *S. alterniflora*, predicting invasion range, and indicating direction. The calculation formula is as follows:

(2) $$\;X_{k}=\frac{\sum_{i=1}^{n}S_{i}x_{i}}{\sum_{i=1}^{n}S_{i}}$$

(3) $$\;Y_{k}=\frac{\sum_{i=1}^{n}S_{i}y_{i}}{\sum_{i=1}^{n}S_{i}}$$

In the above equation, *X*_*k*_ and *Y*_*k*_ represent the centroid coordinates of *S. alterniflora* at different years, where *S_i_* denotes the area of the *i*th *S. alterniflora* patch within the calculation period, and *x*_*i*_ and *y*_*i*_ represent the centroid coordinates of the *i*th *S. alterniflora* patch within the calculation period. The centroid coordinates of the patches are calculated using the “Calculate Geometry” option in ArcGIS, and the centroid coordinates data are then imported into Excel for the computation of the centroid coordinates according to Equations (2) and (3).

With the repeated points in the same grid eliminated by ENMTools to avoid model overfitting, 93 points of *S. alterniflora* distribution were obtained for Maxent model analysis. Comprehensively considering the climatic and geographic characteristics of the growth environment of *S. alterniflora* and selected by Pearson correlation analysis, 16 environmental variables were used as the environmental layer data for analyzing the potential distribution of *S. alterniflora* (Table 2). Environmental variables with a correlation coefficient |*r*| ≥ .75 were subsequently excluded. The retention status of each environmental variable is shown in Table 2. The distribution points for *S. alterniflora* were set with 25% as test samples and 75% as training samples. The repetitive operation type was set to cross‐validation, the number of iterations was set to 10, and the average value was taken as the final prediction result.

**TABLE 2:** List of environment variables.

Variable type	Variable	Month	Description	Unit	Retention status
SST	sst1	11\12\1	Mean sea surface temperature of coldest quarter	°C	√
	sst2	5\6\7	Mean sea surface temperature of warmest quarter	°C	√
	sst3	4\5\6	Mean sea surface temperature of wettest quarter	°C	×
	sst4	9\10\11	Mean sea surface temperature of driest quarter	°C	×
	sst5	—	Annual mean sea surface temperature	°C	√
Climate	climt1	5\6\7	Mean temperature of warmest quarter	°C	√
	climt2	11\12\1	Mean temperature of coldest quarter	°C	×
	climt3	7	Max temperature of warmest month	°C	√
	climt4	12	Min temperature of coldest month	°C	√
	climp1	4\5\6	Mean precipitation of wettest quarter	mm	√
	climp2	9\10\11	Mean precipitation of driest quarter	mm	×
Water quality	sali	—	Monthly range of salinity variation	‰	√
	sali_mean	—	Annual mean salinity	‰	√
Terrain	dem		Elevation	m	√
	slope		Slope	°	√
Soil	soiltype	—	Soil type	—	√

*Note*: Climp data were derived from WorldClim (www.worldclim.com), SST data were derived from Ocean Color SMI: Standard Mapped Image MODIS Terra Data, water quality data were derived from HYCOM: Hybrid Coordinate Ocean Model, Water Temperature and Salinity, Digital Elevation Model (DEM) data were derived from ASTER GDEM (Terra Advanced Spaceborne Thermal Emission and Reflection Radiometer) dataset (http://www.gscloud.cn), and soil data were derived from Resources and Environmental Science Data Center (https://www.resdc.cn).

#### 2.3.5 The importance evaluation method of environmental variables

The Maxent model can provide a numerical reference to the importance of environmental variables. The calculation of percentage contribution takes into account the interaction between environmental variables, while permutation importance represents the effect of a single environmental variable on the model (Phillips, 2010). The contributions of different environmental variables to the analysis results of the Maxent model reflect their impact on the potential distribution of species. Based on the percentage contribution and permutation importance, we identify the environmental factors that have a greater influence on the growth of *S. alterniflora* in the coastal zone of Guangxi.

## 3 RESULTS

### 3.1 Phenological features of *S. alterniflora* extracted by harmonic regression

**FIGURE 4:**
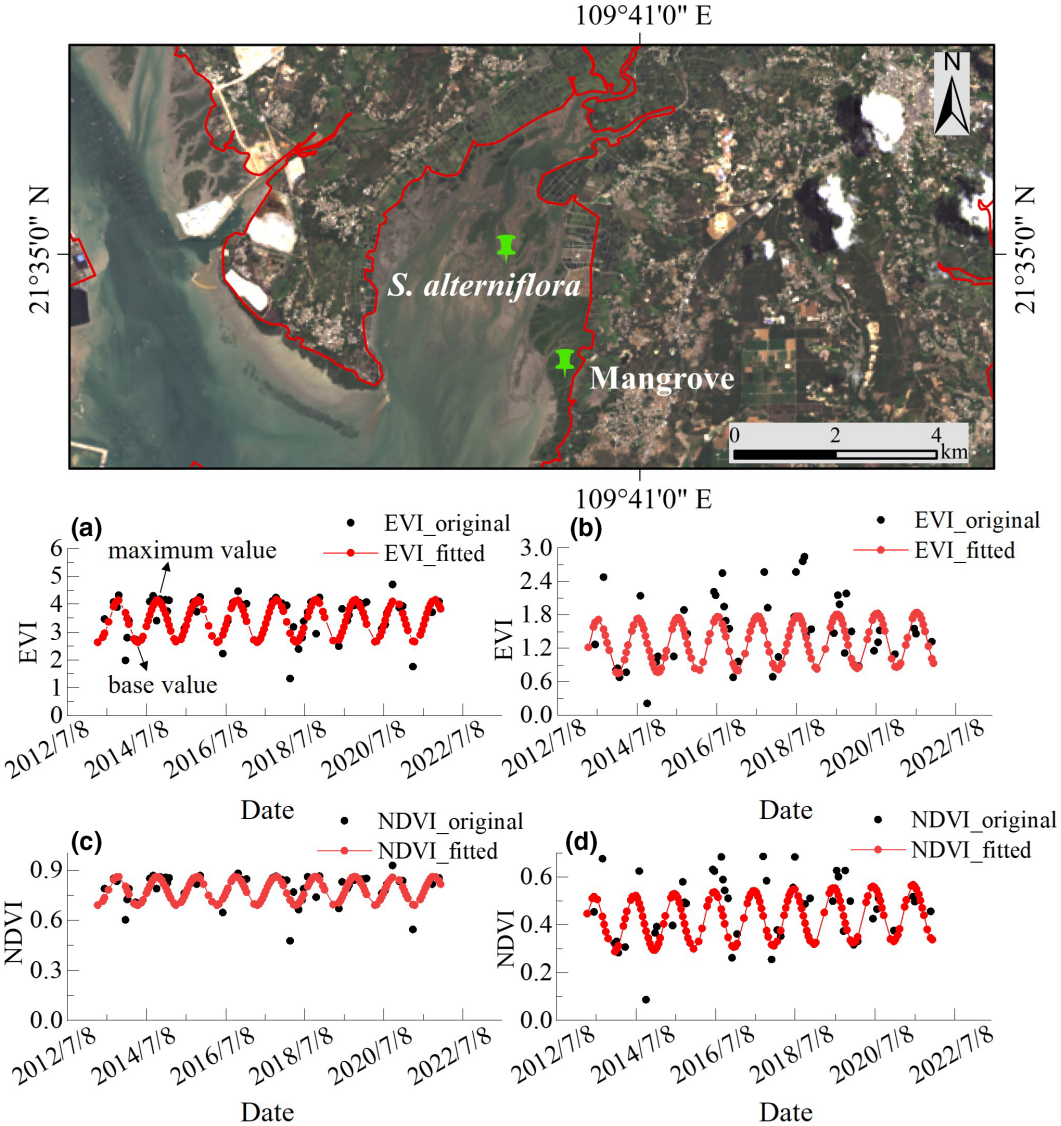
EVI and NDVI time series curves fitted by harmonic regression (a, c) of mangroves; (b, d) of *Spartina alterniflora*.

Mangrove is one of the wetland vegetations growing in the coastal zone of Guangxi, which can be mixed with *S. alterniflora*, and leads to misclassification. Samples of *S. alterniflora* and mangrove were selected to draw EVI and NDVI time series curves, as shown in Figure 4. The change of original data was not obvious and the outliers interfered more, which could not reflect the real growth pattern of the vegetation. Compared with the original time series data, the time series curves fitted by harmonic regression were smoother. The growth pattern of vegetation is clearer with the interference of outliers removed and the trend of main data remains unchanged. The maximum values and base values of the fitted curves of *S. alterniflora* showed a large difference, with the maximum value appeared in May, June, and July, and the base value appeared in November, December, and January of the next year, which is in accordance with the growth pattern of *S. alterniflora*. The EVI and NDVI fitted curves of *S. alterniflora* and mangrove differed in values due to different growth areas and phenological properties. As plants, the EVI and NDVI fitted curves of *S. alterniflora* and mangrove showed high maximum values. However, due to the existence of a senescence period in the growth of *S. alterniflora*, and the fact that mangroves are evergreen shrubs or trees, the EVI and NDVI fitted curves of *S. alterniflora* showed lower base values, while those of mangroves were higher (EVI > 2, and NDVI > 0.6).

**FIGURE 5:**
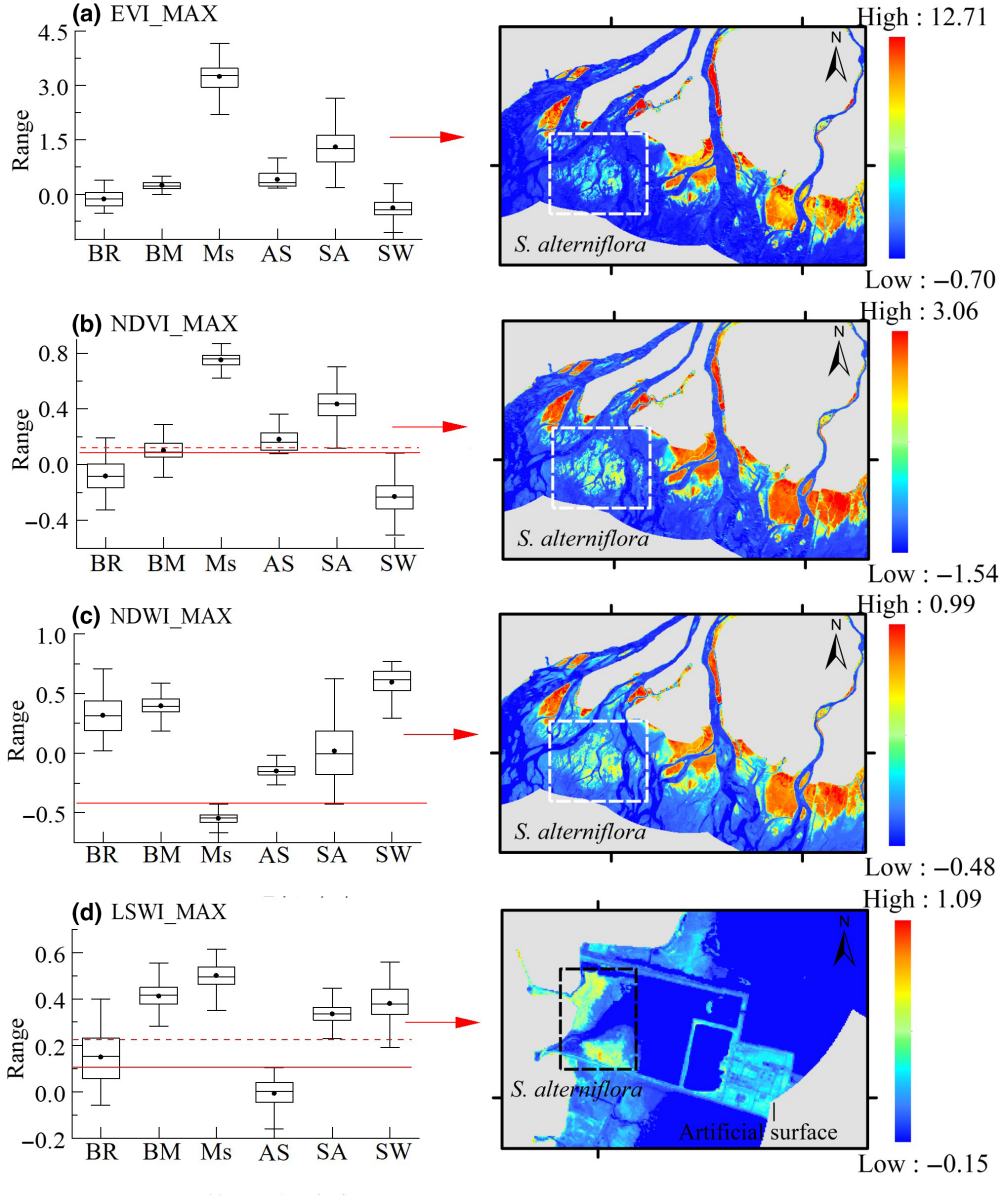
Box diagram of characteristic indices (NDVI, EVI, NDWI, and LSWI) extracted from different ground types, in which BR, BM, Ms, AS, SA, and SW refer to buoyant raft, bare mudflats, mangroves, artificial surface, *Spartina alterniflora*, and seawater, respectively.

According to the box diagram in Figure 5, the maximum value extracted from fitted curves of characteristic indices showed differences in values between different ground objects, proving the phenological features could be extracted to distinguish *S. alterniflora* and non‐*S. alterniflora*.

### 3.2 Accuracy assessment of the *S. alterniflora* maps

1. Accuracy assessments of mapping results with different feature extraction methods.

For the purpose of evaluating the effect of harmonic regression, the maximum values, base values, and variation ranges of characteristic indices were directly extracted from the original data, and the classification of *S. alterniflora* was carried out by random forest with features mentioned above. Results showed that the overall accuracy (OA) was only 50.5% in 2019, and the Kappa coefficient was 0.01. Comparing the classification result with Google Earth images in 2019 (Figure S2), the mapping results of *S. alterniflora* with phenological features extracted from fitted time series data showed a higher accuracy with a relatively appropriate range, while the mapping result of *S. alterniflora* with phenological features extracted from original data showed more misclassification and a much larger range.

2 Accuracy assessments of results in different years.

The accuracy of mapping results of 2009, 2014, and 2019 was evaluated separately (Table 3). Among these, the result of 2009 showed the highest overall accuracy (97.33%). The overall accuracy of 2009, 2014, and 2019 all exceeded 90% and Kappa coefficients exceeded 0.8, indicating that the classification results are credible, and the phenological features perform well in distinguishing *S. alterniflora* in the study area.

**TABLE 3:** Classification results of *Spartina alterniflora.*

Year	Classification accuracy				Area (hm^2^)
	PA	UA	OA	Kappa	
2009	94.94	94.67	97.33	0.95	408.66
2014	83.33	80.00	90.00	0.8	1043.66
2019	88.24	86.67	93.33	0.87	1414.74

Abbreviations: OA, Overall Accuracy; PA, Producer's Accuracy; UA, User's Accuracy.

### 3.3 Evaluation of the Maxent model analysis results

The Receiver Operating Characteristic Curve (ROC) was used to evaluate the analysis results of Maxent models (Hanley & Mcneil, 1982), with sensitivity as the ordinate and specificity as the abscissa. The value of the Area under the ROC curve (AUC) is commonly used to evaluate the performance of the model (Phillips et al., 2006). The value of AUC ranges from 0 to 1, the higher the AUC value, the higher the accuracy of model analysis, and the more reliable the analysis results. The relation between AUC and analysis results is shown in Table S1. After 10 times of cross operation, the ROC curve of the analysis results is shown in Figure S3. The results showed that the AUC value was 0.913, indicating that the model analysis was excellent and the results were reliable. Simultaneously, the Maxent model could better analyze the potential distribution of *S. alterniflora* under the condition of the linear characteristic function.

## 4 DISCUSSION

### 4.1 Expansion of *S. alterniflora* in coastal zone of Guangxi during 2009–2020

**FIGURE 6:**
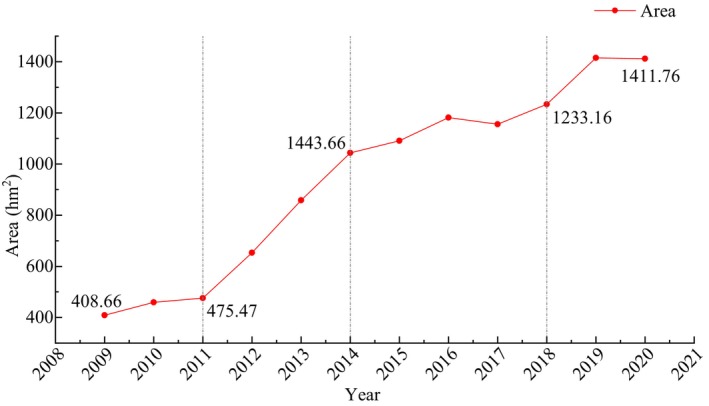
Total area of *Spartina alterniflora* in coastal zone of Guangxi during 2009–2020.

To better understand the expansion of *S. alterniflora* in coastal zone of Guangxi, the mapping area during 2009–2020 was analyzed and is shown in Figure 6. According to the mapping result of 2009, the area of *S. alterniflora* in Dandou Sea was 317.36 hm^2^, which was more consistent with the study result of 301.04 hm^2^ (Li et al., 2021), with a deviation of 5.4%. In the study of (Li et al., 2021), the coverage of *S. alterniflora* was identified by visual interpretation, based on Gaofen‐1 (GF‐1) satellite images (spatial resolution of 2 m in panchromatic image). According to the mapping result of 2018, the total area of *S. alterniflora* in coastal zone of Guangxi was 1233.16 hm^2^, which was consistent with the study result of 1159.21 hm^2^ (Xu et al., 2021), with a deviation of 6.4%. The deviation between the results of this study and those of previous studies was no more than 10%, suggesting that the results are reliable, and the deviation of the mapping results mainly derived from different resolutions of the images.

The area of *S. alterniflora* in coastal zone of Guangxi has been increasing since 2009 (Figure 6). Among these, *S. alterniflora* grew fastest from 2011 to 2014, with annual growth rates higher than 20%. From 2015 to 2019, the trend tended to be gentle, with the area in 2015 only increasing by 4.55% compared with that in 2014. In 2019 and 2020, the area of *S. alterniflora* was close (1414.74 hm^2^ and 1411.76 hm^2^, respectively). Considering the annual growth rate in the past 5 years (average of 5.51%), the *S. alterniflora* in the study area still has a potential for expansion.

The typical distribution regions with highly aggregated patches of *S. alterniflora* in coastal zone of Guangxi include Dandou Sea (Shankou Mangrove Reserve), tidal flat in the estuary of Nanliu River, and the section from Tieshan Port to Yingpan Port. The area of *S. alterniflora* in these three regions is shown in Figure S4.

#### 4.1.1 Coverage changes of *S. alterniflora* in Dandou Sea

Dandou Sea was the first place to introduce *S. alterniflora* in coastal zone of Guangxi and had been continuously suffering the rapid expansion of *S. alterniflora*, which covered 317.36 hm^2^ of area in 2009 (Deng et al., 2020). From 2009 to 2011, changes in the coverage of *S. alterniflora* tended to flatten. From 2012 to 2020, the coverage of *S. alterniflora* showed a trend that increased first, and then slowly decreased, with the area reaching the largest of 536.72 hm^2^ in 2014. From 2016 to 2020, the annual average growth rate of the coverage of *S. alterniflora* was negative (−3.92%), indicating that the growth of *S. alterniflora* in this region was in a saturation period.

**FIGURE 7:**
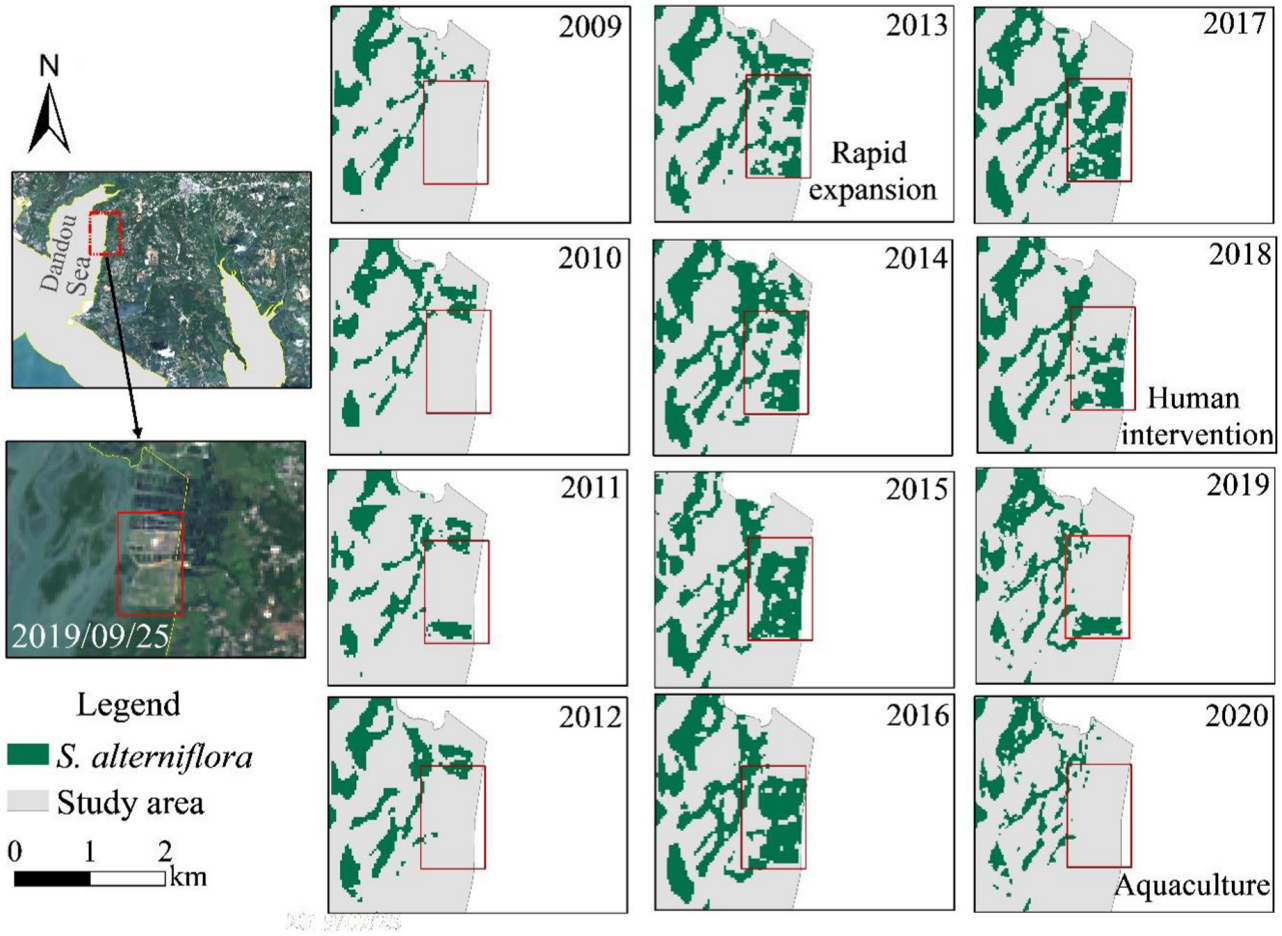
Coverage changes of *Spartina alterniflora* in abandoned ponds during 2009–2020.

The impact of aquaculture on the growth of *S. alterniflora* in Dandou Sea from 2013 to 2020 is shown in Figure 7. The area marked by a red frame was an abandoned pond in 2009, in which *S. alterniflora* continuously expanded during 2013–2017, with an area of 63.02 hm^2^. Since 2018, *S. alterniflora* has been removed due to artificial remediation and gradually restored for aquaculture, and there was no *S. alterniflora* spotted by remote sensing monitoring in this area by 2020. Coverage changes of *S. alterniflora* in the abandoned ponds indicate that human intervention has a certain effect of preventing the spread of the invasive species and reducing the growth of *S. alterniflora*.

**FIGURE 8:**
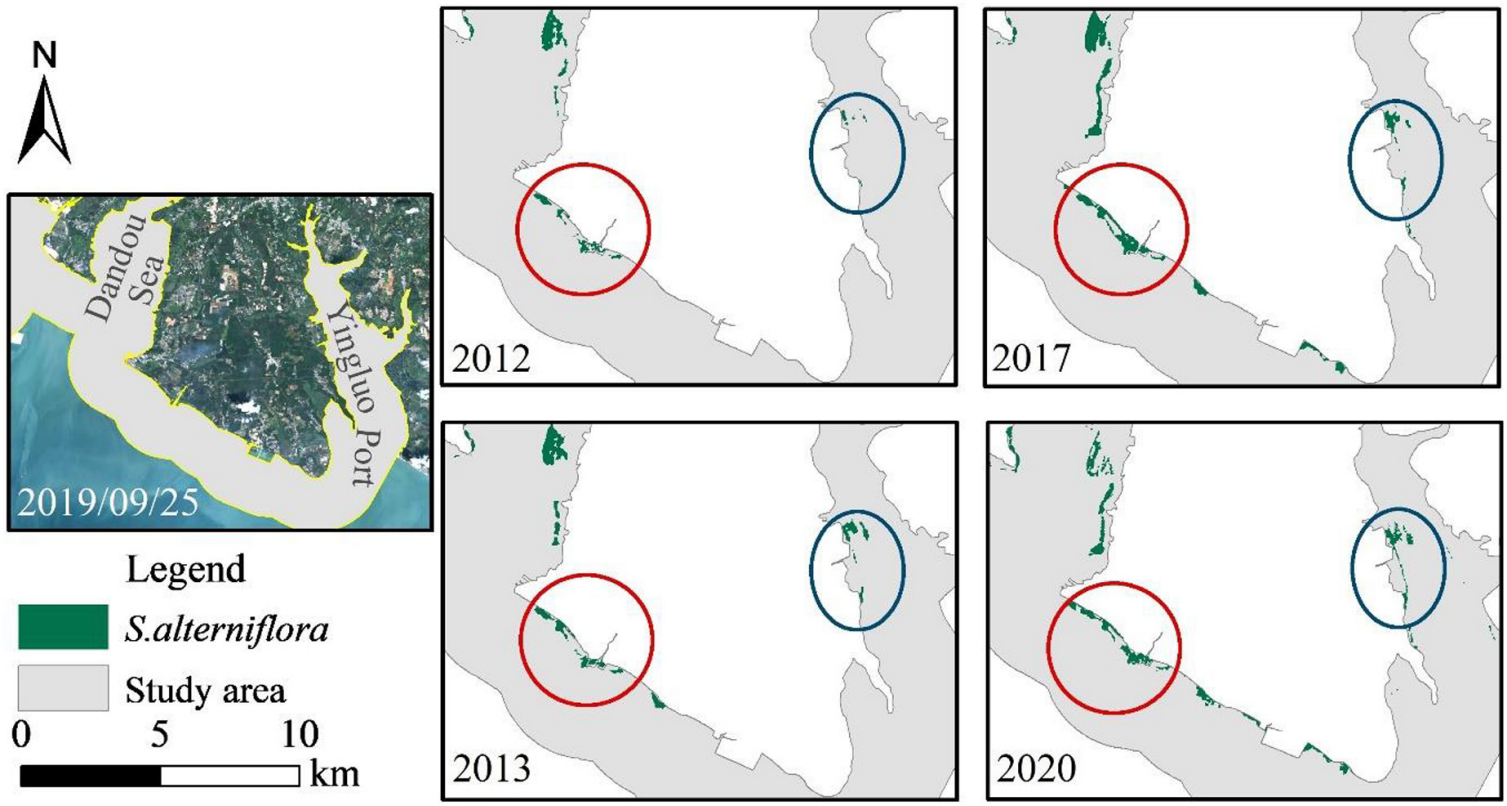
Coverage changes of *Spartina alterniflora* in Yingluo Port during 2012–2020.

Though under similar climatic and topographic conditions, the tidal flat existing from Dandou Sea to the east of Yingluo Port enjoys more sufficient space and less human disturbance. For these reasons, the area of *S. alterniflora* in Yingluo Port increased from 22.4 hm^2^ to 155.14 hm^2^ from 2009 to 2020, showing a trend of expansion (Figure 8).

#### 4.1.2 Coverage changes of *S. alterniflora* in the estuary of Nanliu River

The tidal flat at the estuary of Nanliu River used to be bare mudflats. Due to the suitable climate and sufficient space for growth, the coverage of *S. alterniflora* gradually increased since 2009. The area of *S. alterniflora* in 2011 was 3.93 times that of 2010, and the annual growth rates of coverage from 2012 to 2014 were higher than 55%, indicating the growth of *S. alterniflora* was in a period of rapid expansion. Limited by the reduction of bare mudflats, the growth rate of *S. alterniflora* gradually slowed down from 2015 to 2019, with the growth rate of *S. alterniflora* decreasing to 10.69% in 2019.

**FIGURE 9:**
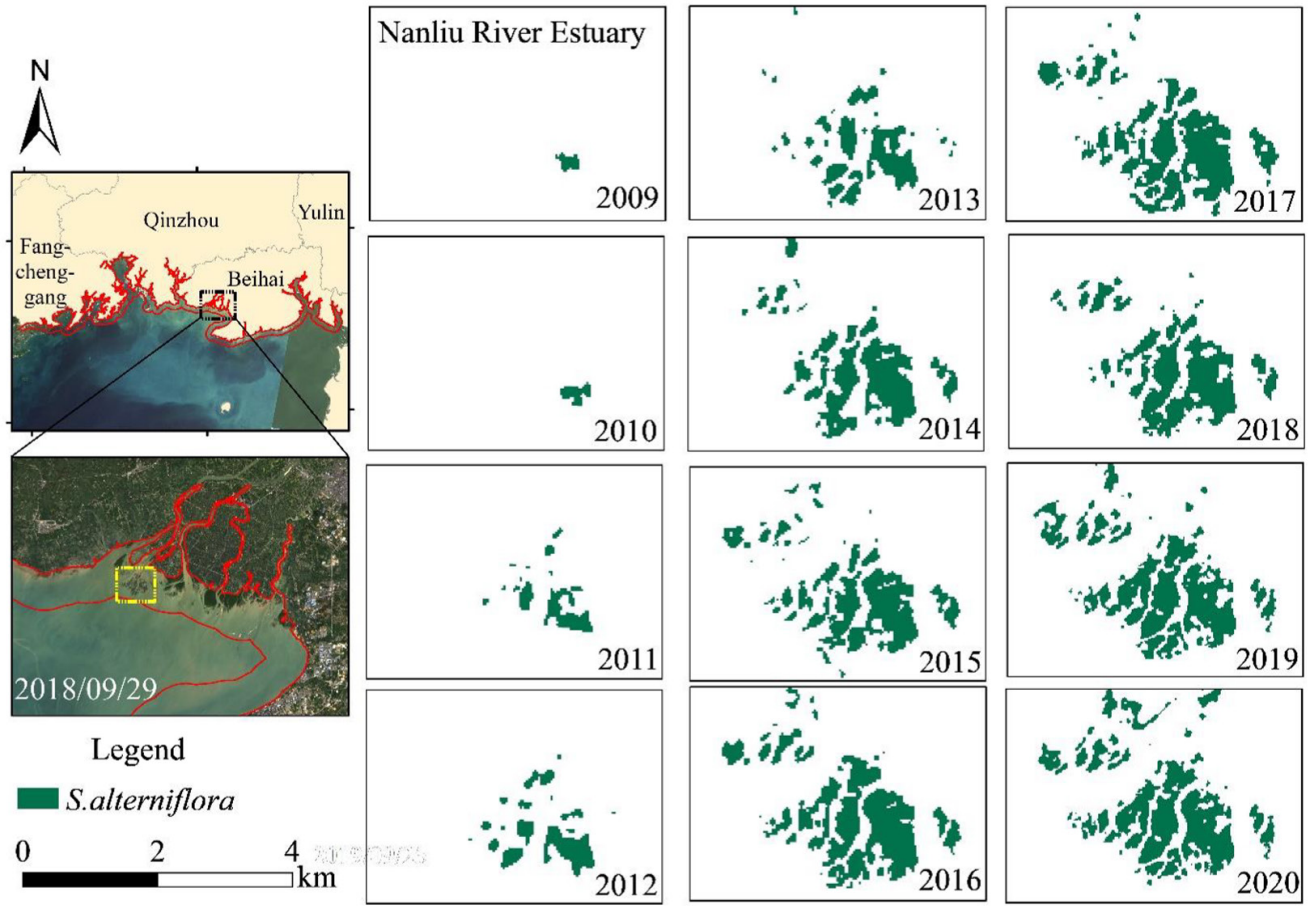
Coverage changes of *Spartina alterniflora* in the estuary of Nanliu River during 2012–2020.

From 2013 to 2020, the density of *S. alterniflora* patches increased significantly. The *S. alterniflora* continuously proliferated to form a dense single‐species community after encroachment on the bare mudflats (Figure 9), which was consistent with the findings of (Pan et al., 2016). Due to the further reduction of expansion space in 2020, the coverage of *S. alterniflora* increased by only 0.25% compared with that in 2019, indicating that the growth of *S. alterniflora* in this region is approaching a period of saturation, which may develop to squeezing of the living space of surrounding wetland vegetation, and expansion to other regions with tidal flats.

**FIGURE 10:**
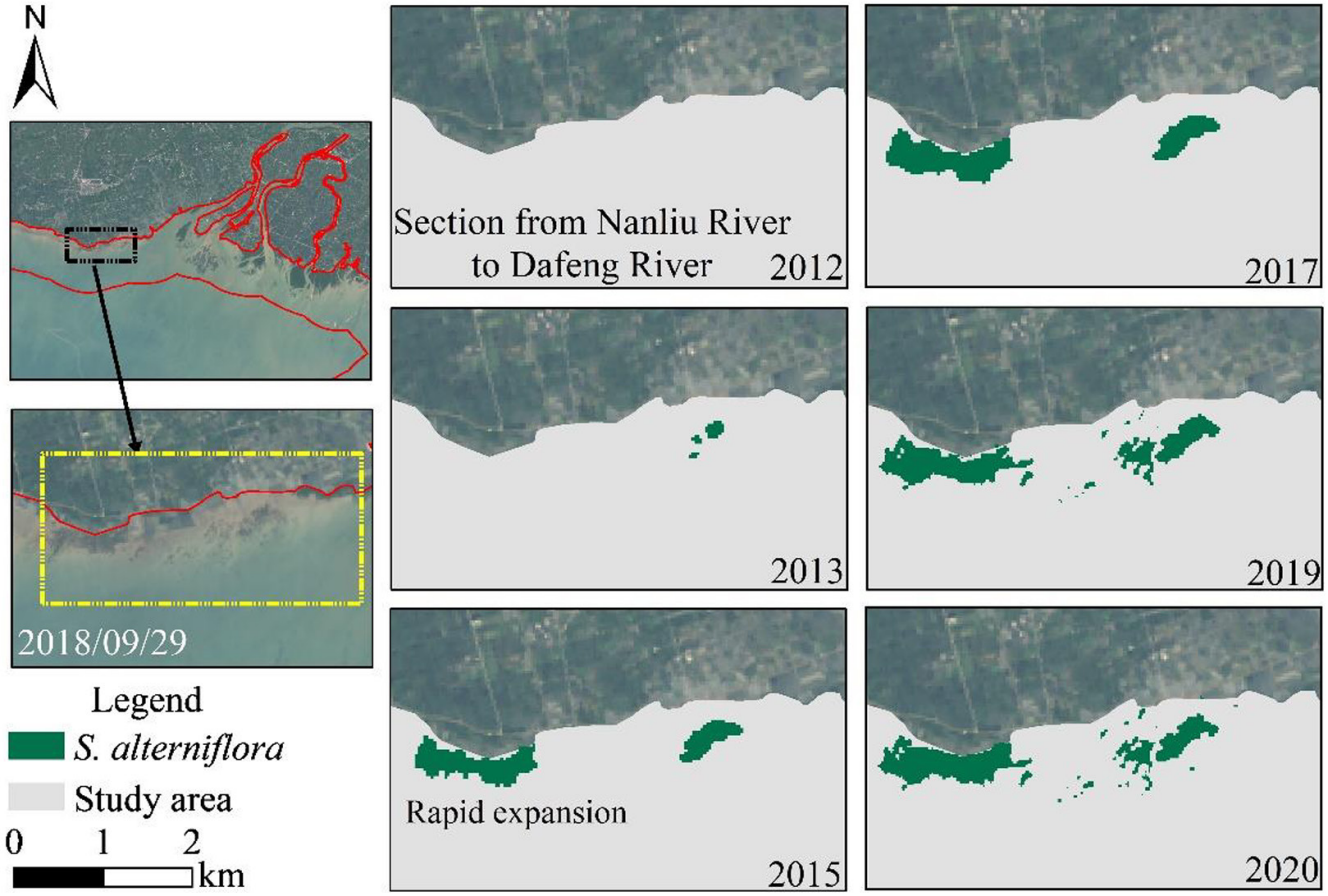
Coverage changes of *Spartina alterniflora* in Dafeng River during 2012–2020.

Some studies have proved that seeds of *S. alterniflora* have a certain fecundity and can spread to other areas with the tide (Deng et al., 2006). *S. alterniflora* was first spotted in the section from Nanliu River to Dafeng River in 2013 and rapidly expanded during 2014–2015 (Figure 10). Then the *S. alterniflora* gradually distributed along Dafeng River in 2019, reflecting the trend of spreading from east to west by tide and currents.

#### 4.1.3 Coverage changes of *S. alterniflora* in Tieshan Port and Yingpan Port

**FIGURE 11:**
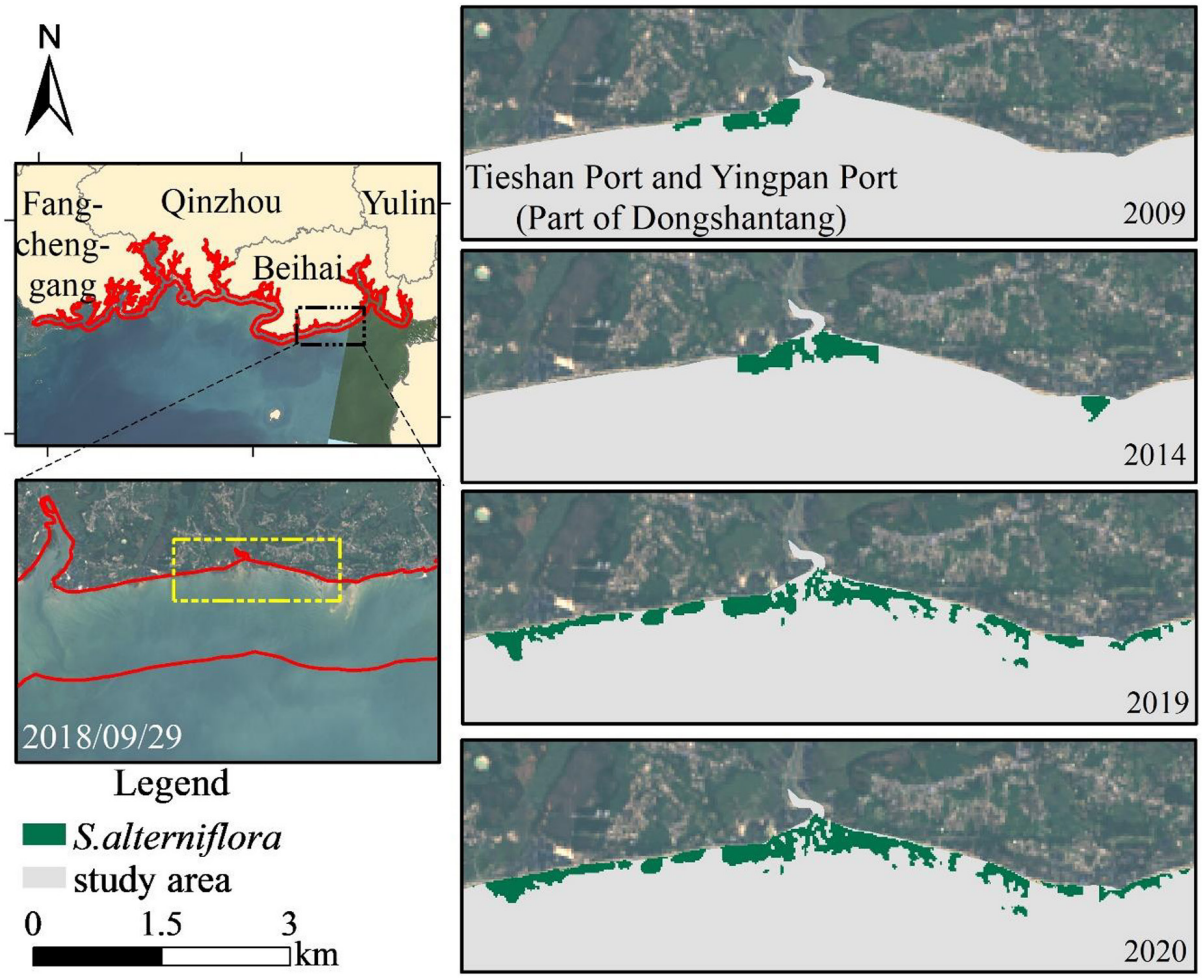
Coverage changes of *Spartina alterniflora* in Tieshan Port and Yingpan Port during 2012–2020.

The coverage of *S. alterniflora* in the coastal area of Tieshan Port and Yingpan Port expanded with the density of patches increasing from 2009 to 2020, with the proliferation rate reaching the highest during 2017–2020, reaching an annual growth rate of 43.68% in 2019. The distribution of *S. alterniflora* in Dongshantang area, located between Tieshan Port and Yingpan Port, is shown in Figure 11. Considering the change of coverage in Tieshan Port and Yingpan Port, the average annual growth rate of *S. alterniflora* in the past 5 years was 16.63%, showing a longitudinal expansion along the coastal area, suggesting the growth of *S. alterniflora* in this region is still in an expansion period, with relatively enough space.

#### 4.1.4 Migration of *S. alterniflora* integral patch centroids during 2009–2020

In terms of the different growth periods of *S. alterniflora* in the three typical regions above, while the coverage decreased in Dandou Sea and the growth rate of *S. alterniflora* decreased in the estuary of Nanliu River, the large expansion of *S. alterniflora* in Tieshan Port and Yingpan Port was the main reason for the small increase of the total area of *S. alterniflora* in coastal zone of Guangxi since 2018. The migration of *S. alterniflora* integral patch centroids from 2009 to 2020 was analyzed, as shown in Figure S5. The coordinates of the integral centroid of *S. alterniflora* patches were obtained based on the area‐weighted calculation of the centroid of each patch, the migration of which reflected the direction of expansion and patch distribution of *S. alterniflora* in the Guangxi coastal zone. From 2009 to 2020, the integral centroid of *S. alterniflora* migrated by 17,858.7 m from east to west, indicating that *S. alterniflora* spread from east to west in the study area. The location of the centroid shifted from Dandou Sea to Tieshan Port and Yingpan Port, indicating that *S. alterniflora* grew actively and patches aggregated in Tieshan Port and Yingpan Port, proving a high potential for expansion.

### 4.2 Potential distribution analysis of *S. alterniflora*

#### 4.2.1 Analysis on potential distribution conditions of *S. alterniflora*

The analysis results of the contribution and importance of environmental variables calculated by the Maxent model are shown in Table 4. Taking sst1 as an example, this environmental variable has the highest percentage contribution and permutation importance, reaching 27.5% and 25.2%, respectively, demonstrating that sst1 has the most significant impact on the model analysis results and is less affected by variable interactions. Analyzing the contribution and importance of different environmental variables to the model results can reflect their degree of influence on the species' habitat suitability. According to Table 4 and Figure S6, five environmental factors are significantly associated with the growth of *S. alterniflora* in the coastal zones of Guangxi. These variables include coastal saline soil, sst1, sst2, sst5, and climp1.

**TABLE 4:** Percentage contribution and permutation importance of the variables.

Variable	Percentage contribution (%)	Permutation importance (%)
sst1	27.5	25.2
slope	21.3	7
sst5	18	7.7
soiltype	10.1	3.8
climp1	6.9	15.5
sst2	6	16.5
sali_mean	3.5	10.2
sali	3.1	1.3
climt4	2.3	3.2
climt1	0.6	1.3
climt3	0.5	7.7
dem	0.1	0.4

**FIGURE 12:**
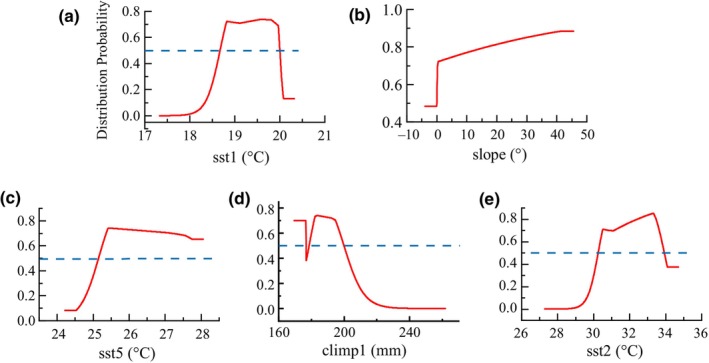
Response curve of dominant environmental factors.

Based on the output result of the Maxent model, the distribution probability of *S. alterniflora* under different environmental conditions is shown in Figure 12. The response curves of sst1 and sst2 indicate that the probability distribution of *S. alterniflora* exhibits an increasing trend followed by a decreasing trend. When sst1 was 18.67 ~ 20.01°C and sst2 was 30.26 ~ 33.93°C, the probability distribution of *S. alterniflora* was more than 0.5. The sst5 response curve revealed that the distribution probability of *S. alterniflora* was higher than 0.5 when sst5 was higher than 25.15°C. When climp1 was 183.0 mm, the distribution probability of *S. alterniflora* reached the maximum, then the distribution probability decreased with the increase of climp1, and the response curve showed a downward trend. According to the response curve of slope, the higher the slope, the higher the probability of distribution of *S. alterniflora* (always above 0.5).

**FIGURE 13:**
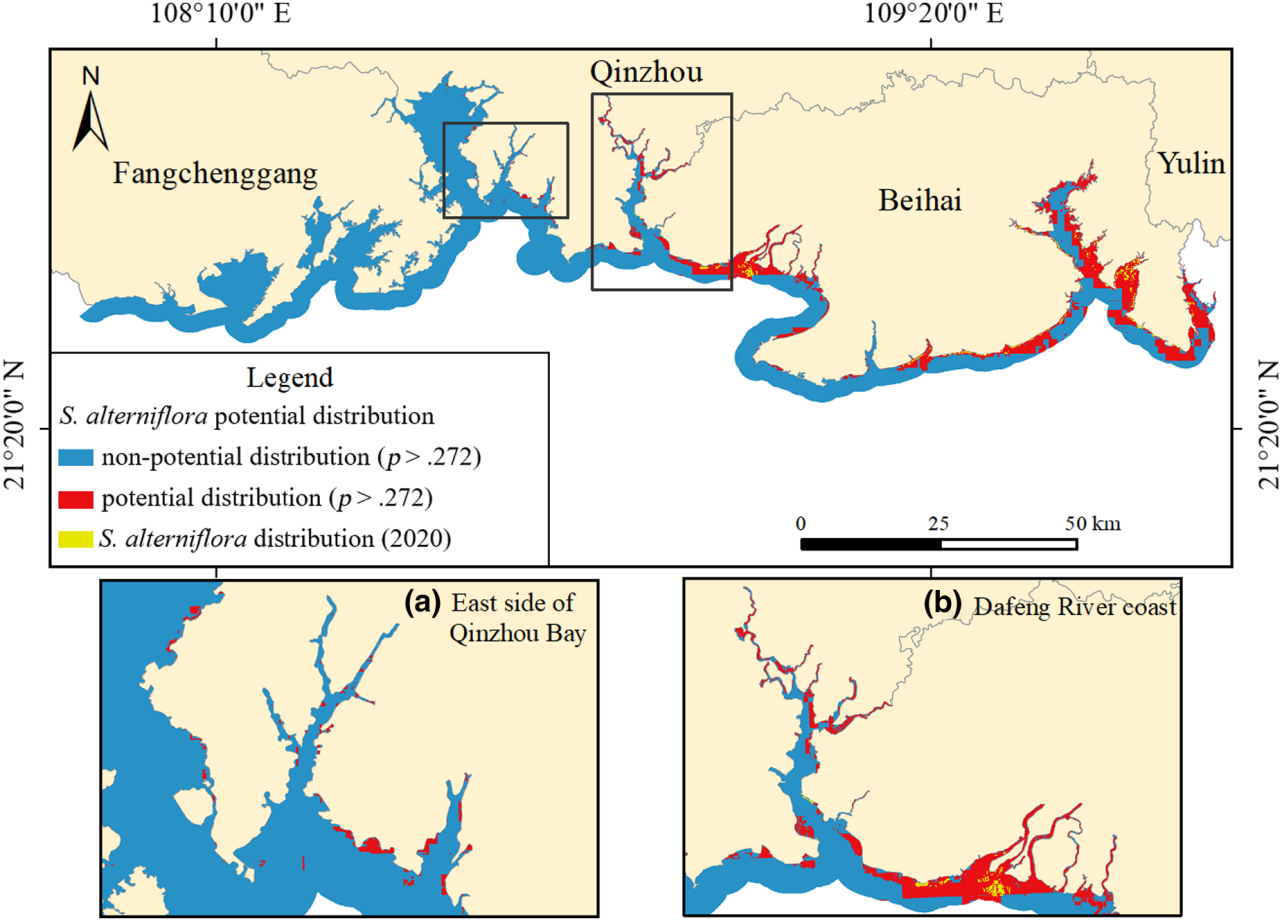
Potential distribution of *Spartina alterniflora* in coastal zone of Guangxi.

The ability of *S. alterniflora* seeds to travel great distances on the tides is a major characteristic that contributes to the plant's effective invasion and rapid expansion (Domènech & Vilà, 2008). Studies have proven that the germination rate of *S. alterniflora* seeds increases with increasing temperature (Li et al., 2019; Wang, Zhou, et al., 2021; Zhu et al., 2011). Furthermore, high salinity conditions inhibit the germination of seeds from *S. alterniflora*. Sufficient precipitation can promote the sexual reproduction of *S. alterniflora* by reducing salt stress, but excessive precipitation can also cause waterlogging stress. The response curve (Figure 13) showed that the average precipitation in the wettest season maintains a high distribution probability to a certain extent, and the distribution probability decreases with the increase of precipitation beyond the suitable range.

According to the results of the response curve analysis, the most suitable environmental conditions for *S. alterniflora* growth were coastal saline soil, sst1 ranged from 18.67 to 20.01°C, sst2 ranged from 30.26 to 33.93°C, sst5 greater than 25.15°C, and climp1 less than 178.59 mm.

#### 4.2.2 Classification of potential distribution area for *S. alterniflora*

Taking the maximum sum of sensitivity and specificity of training samples as the threshold, the potential distribution region (distribution probability >0.272) and non‐potential distribution region (distribution probability <0.272) of *S. alterniflora* were divided. According to the results, the potential distribution of *S. alterniflora* in the coastal zone of Guangxi covered a total area of 24,667.85 hm^2^. The main potential distribution regions were Dafeng River, Nanliu River Estuary, Tieshan Port to Yingpan Port, and Dandou Sea (Figure 13), which were consistent with the existing distribution areas of *S. alterniflora*.

The existing distribution area of *S. alterniflora*, which is divided into areas of highly potential distribution, has the highest possible distribution probability. High crown density mangrove environments make it difficult for *S. alterniflora* to invade, and more likely to expand to the sea and occupy the beaches. In this case, mangrove areas and tidal flats were divided into areas of least and moderate potential distribution, as shown in Table 5. According to the results of the Maxent model, the Guangxi coastal zone had a potential growth distribution area of 14,303.39 hm^2^ for *S. alterniflora*, with highly, moderately, and least potential distribution regions accounting for 9.87%, 71.56%, and 18.57% of the total potential distribution regions of *S. alterniflora*, respectively.

**TABLE 5:** Potential area of *Spartina alterniflora* in different regions.

Name	Highly potential distribution (hm^2^)	Percentage (%)	Moderately potential distribution (hm^2^)	Percentage (%)	Least potential distribution (hm^2^)	Percentage (%)
Guangxi Coastal Zone	1411.76	9.87	10,235.01	71.56	2656.62	18.57
Dandou Sea	399.49	19.47	1206.67	58.81	445.57	21.72
Yingluo Port	155.14	7.61	1290.07	63.30	592.74	29.09
Tieshan Port to Yingpan Port	541.63	8.74	4861.99	78.47	791.99	12.78
Tidal flat of the Nanliu River Estuary	204.93	7.07	2239.41	77.26	454.19	15.67
Dafeng River and Qinzhou Gulf	110.57	9.88	636.87	56.89	372.13	33.24

#### 4.2.3 Potential distribution and control suggestions for *S. alterniflora* on the Guangxi coastal zone

Dandou Sea is located in Shankou Mangrove Reserve. Studies have shown that *S. alterniflora* inhibited the growth and expansion of mangroves from 2005 to 2008, causing a gradual decline in mangrove areas in the region (Shen et al., 2022). Mixed growth phenomena of *S. alterniflora* and mangroves exist in the Dandou Sea area, with space for *S. alterniflora* to expand into the sea. Therefore, it is suggested that the high and least potential distribution of *S. alterniflora* should be controlled simultaneously.

**FIGURE 14:**
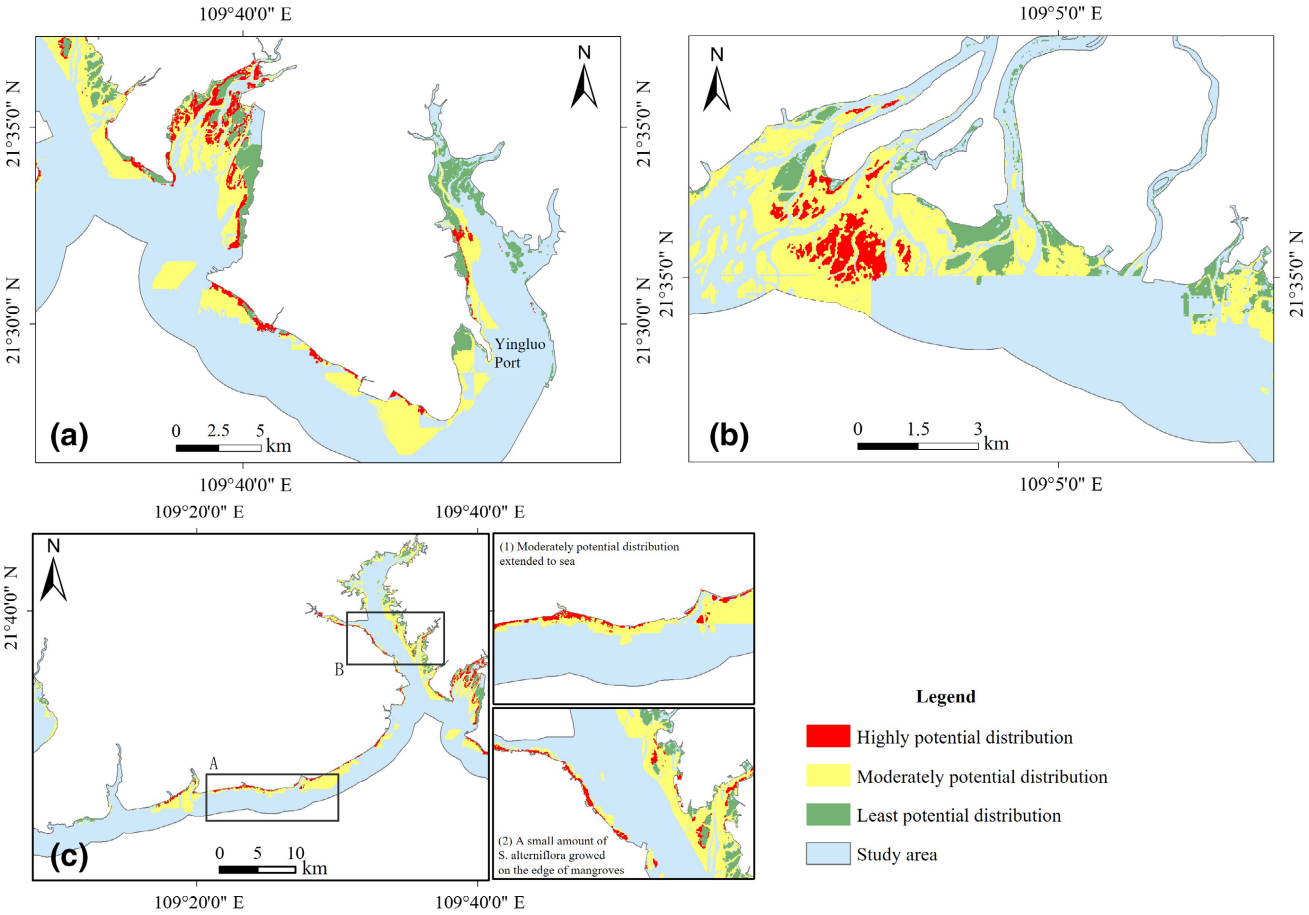
Potential distribution of *Spartina alterniflora* in coastal zone of Guangxi; (a) Dandou Sea, (b) Nanliu River, and (c) Yingpan Port.

The high potential distribution of *S. alterniflora* in the tidal flat of the Nanliu River Estuary reached 204.93 hm^2^, accounting for 7.07% of the total potential distribution area of the region (Figure 14). The growth of *S. alterniflora* was dominated by independent patches with concentrated distribution, and mangroves in the area grow densely, making it a challenge for *S. alterniflora* to invade. Therefore, recommendations for this region, and the treatment of highly potential distribution, should be given prior consideration, and *S. alterniflora* should be cleared through physical and chemical methods.

From Tieshan Port to Yingpan Port, *S. alterniflora* was in a period of rapid expansion, with high potential distribution accounting for 8.74% of the potential distribution, whose growth range was laterally distributed in coastal tidal flats. Among them, the moderately potential distribution covered the widest area of 4861.99 hm^2^, accounting for 78.47% of the total potential distribution area of *S. alterniflora* in the region (Figure 14). Mangroves had high crown density with the least risk of invasion by *S. alterniflora*. Consequently, it is suggested that the management of highly potential distribution and the prevention of moderately potential distribution should be the main measures, realizing the elimination of *S. alterniflora* and prevention of its expansion by human intervention.

The areas of the Dafeng River and the Qinzhou Gulf were dominated by a moderate potential distribution of *S. alterniflora*, which accounted for 56.89% of the total potential distribution area of the region. According to the mapping results, *S. alterniflora* showed an expanding trend from east to west in the Guangxi coastal zone. The eastern side of Qinzhou Gulf was divided into *S. alterniflora* possible distribution, even though no *S. alterniflora* was detected there because of its regional climate and topography, which carried a risk of invasion. Therefore, it is suggested that *S. alterniflora* invasion should be mainly prevented in this region, and *S. alterniflora* can be detected regularly and cleaned in time to reduce the possibility of a large‐scale invasion.

### 4.3 Shortcomings and future prospects of this study

Due to the time series nature of the study, we used Landsat imagery as the data source. However, it is not possible to accurately identify small patches of newly grown *S. alterniflora* because of its limited spatial resolution. And there might be a problem of misclassification of some native salt marsh vegetation. In future research, the interpretation accuracy of *S. alterniflora* can be improved by integrating multi‐source imagery or combining optical imagery with radar remote sensing data. Additionally, considering the growth cycle as a classification feature can further differentiate *S. alterniflora* from local salt marsh vegetation.

Previous studies have proven that Maxent performed well in identifying invasive species, even with low sample sizes. Our results demonstrated that *S. alterniflora* had an obvious trend of westward expansion in the coastal zone of Guangxi, and had a wide potential distribution area. The results achieved by the Maxent model in the region indicated its robustness, which is essential for *S. alterniflora* studies and management. Due to data source limitations, the current analysis only considers precipitation, temperature, topography, and salinity‐type data as environmental variables. Since *S. alterniflora* thrives in the intermediate–high tide zone and disperses over long distances through tidal flow, its growth is influenced by water quality. In future studies, it would be beneficial to include intertidal elevation data, tidal flow direction and velocity data, wind direction data, and speed data, as well as water quality data as additional environmental variables to further improve the accuracy of the predictive model.

## 5 CONCLUSIONS

Limited by image quality and time of image acquisition, mapping *S. alterniflora* by a single image may cause misclassification and omission. Our result shows that: The harmonic regression exhibits superior accuracy and Kappa coefficient. From 2009 to 2020, the total area of *S. alterniflora* in coastal zone of Guangxi showed a trend that increased first and then decreased, the area of *S. alterniflora* was 1411.76 hm^2^ in 2020. The integral centroid migrated from east to west, shifting from Dandou Sea to Tieshan Port and Yingpan Port. This indicates highly aggregated patches of *S. alterniflora* in Tieshan Port and Yingpan Port, confirming the high potential for expansion in this region. Due to human intervention and natural conditions, *S. alterniflora* in different regions of the Guangxi coastal zone are in different growth stages. In the analysis results of the Maxent model, the degrees of potential distribution differed in different regions. It is necessary to put forward corresponding control suggestions for each level of potential distribution, which will be more conducive to the protection of local biodiversity. By mapping the coverage and analyzing the growth pattern of *S. alterniflora* in coastal zone of Guangxi, the results provide a scientific basis for controlling the expansion of *S. alterniflora* and improving the management of the wetland ecosystem in coastal zone of Guangxi.

## AUTHOR CONTRIBUTIONS

**Huanmei Yao:** Methodology (equal); resources (equal); supervision (equal); writing – review and editing (lead). **MeiJun Chen:** Formal analysis (equal); validation (equal); visualization (equal); writing – original draft (equal); writing – review and editing (equal). **Zengshiqi Huang:** Conceptualization (equal); data curation (equal); methodology (equal); writing – original draft (equal). **Yi Huang:** Conceptualization (equal); formal analysis (equal); methodology (equal); software (equal); writing – original draft (equal). **Mengsi Wang:** Methodology (equal); visualization (equal); writing – review and editing (equal). **Yin Liu:** Visualization (equal); writing – review and editing (equal).

## FUNDING INFORMATION

None.

## CONFLICT OF INTEREST STATEMENT

The authors do not have any competing interests to declare.

## Supporting information


Appendix S1.

Appendix S2.

## Data Availability

All the raw data supporting this work are provided as Appendices S1 and S2.
