# Supplementary material for: Remote sensing monitoring and potential distribution analysis of Spartina alterniflora in coastal zone of Guangxi
Source: Ecol Evol. 2024 May 31;14(6):e11469. doi: 10.1002/ece3.11469 (PMC11140692; doi:10.1002/ece3.11469)
Supplement: Supplementary file 1 — Appendix S1. [file ECE3-14-e11469-s001.docx]

**Fig.S1 Available pixels of Landsat 7/8 images in the study area from 2009 to 2020**

Fig.S2 Comparison of mapping results of *S. alterniflora* in 2019 with feature extracted by harmonic regression fitting(a1/b1), and feature extracted directly(a2/b2)


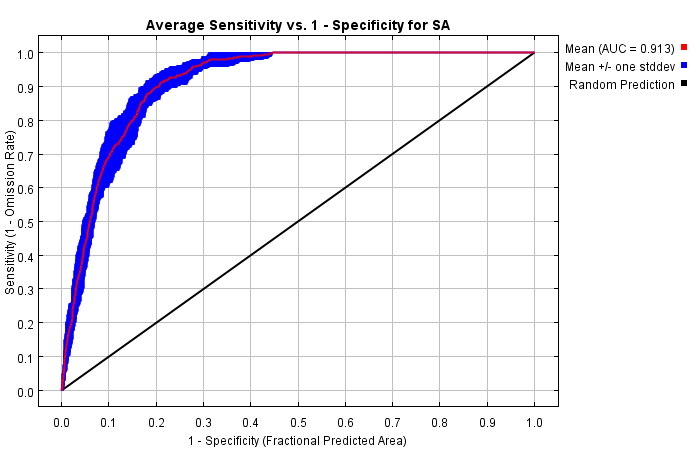


Fig.S3 ROC curve of Maxent model

Fig.S4 Area of *S. alterniflora* in typical distribution regions during 2009-2020

Fig.S5 Centroid changes of *S. alterniflora* during 2009-2020


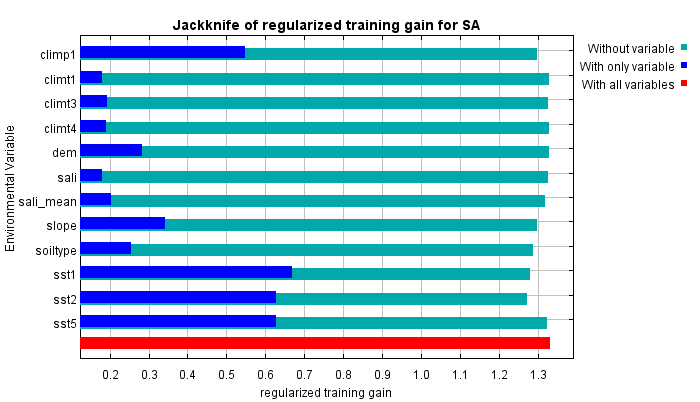


Fig.S6 Jacknife plot of regularized training gain

**Table S1 The relation between AUC and analysis effects**

| **AUC** | **Analysis effects** |
| --- | --- |
| 0.5~0.6 | Failure |
| 0.6~0.7 | Worse |
| 0.7~0.8 | General |
| 0.8~0.9 | Better |
| >0.9 | Excellent |
